# Post-trial follow-up methodology in large randomized controlled trials: a systematic review protocol

**DOI:** 10.1186/s13643-016-0393-3

**Published:** 2016-12-15

**Authors:** Rebecca Llewellyn-Bennett, Louise Bowman, Richard Bulbulia

**Affiliations:** Clinical Trial Service Unit, Nuffield Department of Population Health, University of Oxford, Richard Doll Building, Roosevelt Drive, Oxford, OX3 7LF UK

**Keywords:** Methodology, Post-trial, Retention, Randomized controlled trial, Long term, Cost, Follow-up, Effective

## Abstract

**Background:**

Clinical trials typically have a relatively short follow-up period, and may both underestimate potential benefits of treatments investigated, and fail to detect hazards, which can take much longer to emerge. Prolonged follow-up of trial participants after the end of the scheduled trial period can provide important information on both efficacy and safety outcomes. This protocol describes a systematic review to qualitatively compare methods of post-trial follow-up used in large randomized controlled trials.

**Methods/design:**

A systematic search of electronic databases and clinical trial registries will use a predefined search strategy. All large (more than 1000 adult participants) randomized controlled trials will be evaluated. Two reviewers will screen and extract data according to this protocol with the aim of 95% concordance of papers checked and discrepancies will be resolved by a third reviewer. Trial methods, participant retention rates and prevalence of missing data will be recorded and compared. The potential for bias will be evaluated using the Cochrane Risk of Bias tool (applied to the methods used during the in-trial period) with the aim of investigating whether the quality of the post-trial follow-up methodology might be predicted by the quality of the methods used for the original trial.

**Discussion:**

Post-trial follow-up can provide valuable information about the long-term benefits and hazards of medical interventions. However, it can be logistically challenging and costly. The aim of this systematic review is to describe how trial participants have been followed-up post-trial in order to inform future post-trial follow-up designs.

**Systematic review registration:**

Not applicable for PROSPERO registration.

**Electronic supplementary material:**

The online version of this article (doi:10.1186/s13643-016-0393-3) contains supplementary material, which is available to authorized users.

## Background

Randomized controlled trials (RCTs) are considered to be the gold standard for assessing the effects of a treatment. However, RCTs are costly and usually involve a relatively brief treatment period with limited follow-up. A treatment response restricted to this brief “in-trial” period can potentially underestimate the long-term benefits of treatment and also may fail to detect delayed hazards.

Post-trial follow-up (PTFU) is defined here as extended follow-up which starts after the end of the scheduled period of the original trial. Longer term follow-up of trial participants is important as persistent effects may be detected years later after treatment cessation [1] or even enhanced benefits observed decades later—a so-called “legacy-effect” [2]. Furthermore, delayed hazards may only emerge several years after exposure to certain treatments. Therefore, PTFU may add significant scientific value to the evaluation of many healthcare interventions.

There is a wide literature describing the importance of completeness of follow-up during the in-trial period of a RCT, without which the unbiased ascertainment of outcomes may be compromised and statistical power considerably reduced [3]. Many strategies to enhance follow-up during RCTs have been investigated and this remains an area of much ongoing research [4]. Without high quality in-trial follow-up, the value of post-trial follow-up will be extremely limited.

By contrast, little research has been done to evaluate methods for PTFU. Face-to-face follow-up is widely used during the initial "in-trial" period, but is costly if employed longer term. Telephone-based approaches are more practical, with the ability to contact many participants coordinated by a central trial office, and postal follow-up has been shown to be effective [1]. Web-based techniques may become more widespread as technological advances develop [5].

The use of routine health records can provide detailed information relatively inexpensively [6], but the availability of such data and rules governing access to it varies across countries. In the UK, Health Episode Statistics (HES) are held by the Health and Social Care Information Centre (HSCIC) and can be used as a streamlined method to follow-up trial participants. These routinely collected electronic health records include diagnostic codes (ICD-10) for hospital admissions and can be supplemented with mortality records and cancer registry data.

## Methods/design

### Eligibility criteria

#### Study designs

All published, health-related RCTs which have recruited more than 1000 participants and implemented PTFU are to be included in this systematic review. The RCT must have reached its scheduled end before PTFU commenced. Only studies published between 2006 and 2016 will be included.

Health-related interventions will include medical (licensed or unlicensed drugs), surgical, or psychological treatments. There will be no time limit of post-trial follow-up (Table 1).

**Table 1:** Selection criteria of published articles eligible for systematic review

Criteria	Variables
Inclusion criteria	• Large (>1000 participants) randomized controlled trials only • Randomized controlled trials in adult humans • Any type of methodology used for post-trial follow-up • Healthcare intervention for the purpose of treatment • Published articles
Exclusion criteria	(*a*) Publication type • Narrative reviews • Editorials • Commentaries • Unpublished manuscripts • Dissertations • Government reports • Books and book chapters • Conference proceedings • Lectures and addresses • Consensus development statements (including guideline statements)
	(*b*) Study design • Non-randomized studies
	(*c*) Study population • Animals • Children

#### Participants

Trials including participants aged over 18 years old are eligible.

#### Interventions

Methods and incentives (monetary or by other means) used for post-trial follow-up including direct “face-to-face” follow-up and indirect follow-up, eg, medical record review, telephone and postal follow-up, and electronic follow-up including access to electronic health records will be included.

#### Comparators

Methodology used to follow up participants’ post trial will be compared qualitatively in a table format.

#### Outcome measures

Included studies must have published the total number of participants followed-up compared to the total number alive at the end of the in-trial period to calculate retention rates. Where available, secondary outcome measures of cost, incentives used for follow-up, and cost-effectiveness will be recorded and assessed. If there are missing data, an attempt to contact the study authors will be made. Further exploratory comparisons will be made depending on the information available (for example, describing the use of different approaches according to context, such as regional variations or comparisons of industry-funded trials versus those funded through other sources).

#### Language

Only studies published in English will be included.

### Search methods

#### Electronic searches

The electronic search strategy includes the last 10 years of published articles using broad search criteria (Appendix). Searches for eligible studies will take place in a structured, step-wise process. A screening log will be kept. Results of searches from each electronic database and registries will be logged. The following electronic databases will be searched:

- Cochrane methodology group register
- Cochrane Central Register of Controlled Trials (CENTRAL)
- MEDLINE®
- EMBASE

Other sources of searches will include the following trials registry:

- Trials registry: Clinical-trials.gov (http://clinicaltrials.gov/)

#### Screening for eligible studies

**Fig. 1:**
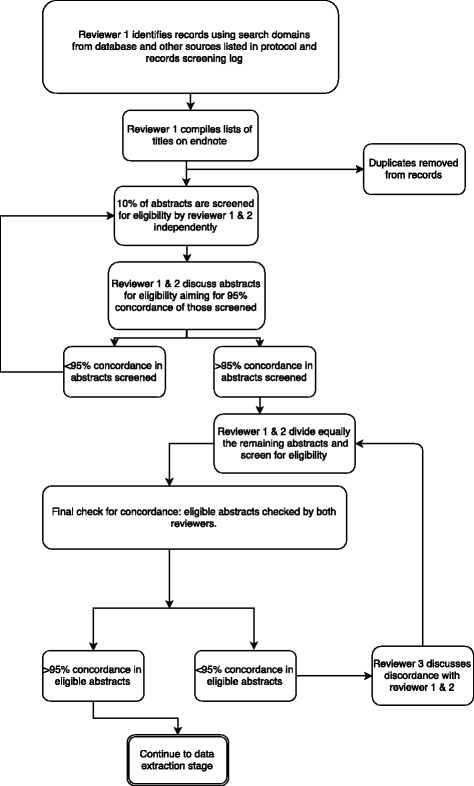
Process of screening abstracts and checking for concordance between reviewers

One reviewer will compile the titles and abstracts of all citations retrieved from the electronic database searches and order these by record number in Endnote® reference management software. Duplicates will be removed using the “deduplication tool” [7]. The screening process will involve two reviewers. The first 10% of abstracts will be screened by both reviewers independently. Concordance of 95% between both reviewers’ decisions on screening will be sought. If concordance is not reached at this point, discrepancies will be discussed and reviewed (including consultation with a third reviewer if necessary), and a further 10% of abstracts will be reviewed (Fig. 1). Once concordance has been reached, the remaining records for screening will be shared equally between the two reviewers and abstracts will be checked for eligibility. All records that are considered to be eligible will be confirmed by both reviewers. Full-text papers will be requested for all potential eligible papers.

### Data collection and analysis

#### Data extraction and management

**Fig. 2:**
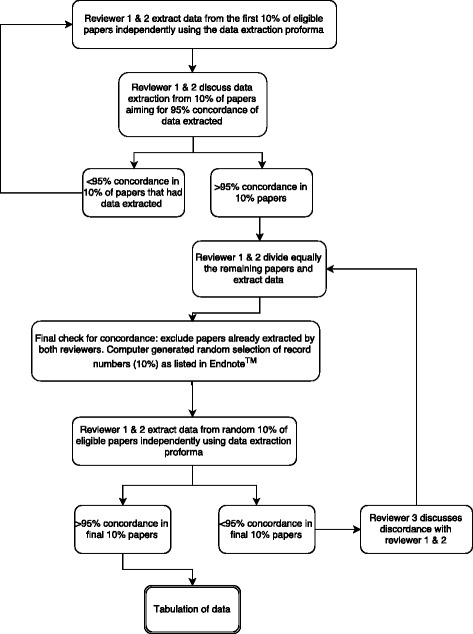
Process of extracting data and checking for concordance between reviewers

Two reviewers will follow a similar step-wise process for data extraction (Fig. 2). A data extraction form will be used, and data extracted from all eligible studies will be compared qualitatively. All data regarding the intervention, the participants (demographics), attrition, retention, incentives used, and if specified, costs of PTFU will be extracted. If required data items are not available in the published article, the study’s corresponding authors will be contacted. If no response is received after two further attempts or from an alternative contact, the study will be excluded from the analysis but recorded on the PRISMA diagram and in an appendix.

#### Assessing the quality of the post-trial follow-up methodology

In order to investigate whether the quality of the post-trial follow-up methodology might be predicted by the quality of the methods used for the original trial, risk of bias will be assessed in those trials chosen for data extraction using the Cochrane Risk of Bias tool. The tool will be applied to the methods used in the main trial, (not the PTFU) focusing on incomplete data; outcome reporting; for-profit bias and other bias sources. Two reviewers will independently assess the risk of bias, and disagreements will be resolved by a third reviewer. The assessment of bias results will be taken into account as part of the assessment of quality of the PTFU methods used.

#### Presenting and reporting of results

The Preferred Reporting Items for Systematic Review Protocols (PRISMA-P) [8] will be followed, including a PRISMA diagram to illustrate the process of selecting eligible studies (Fig. 1). Using the PRISMA guidelines (Additional file 1), the results of this review will be presented and the outcomes tabulated with respect to the different methodologies used in a qualitative and comparative style.

#### Interpretation of findings

The findings of this review will be discussed and potential limitations considered.

## Discussion

Large randomized trials are essential for determining the magnitude of the effects of an intervention. Post-trial follow-up of large RCTs is important, not only for defining the effect of an intervention long-term but also for ascertaining the safety profile and potential hazards which might not be apparent during the relatively brief in-trial period. However, randomized trials can be very expensive, and funding is limited, hence streamlined and effective methodology for PTFU is desirable. This systematic review aims to inform the design of post-trial follow-up for a wide range of randomized trials.

## Appendix

### Search strategies

#### MEDLINE search strategy

- Search conducted via OvidSP interface: 1946 to present in process and other non-indexed citations

**Table:**

Step	Search domain	Search terms
1	Randomized controlled trials	randomized controlled trial.pt. or random?ed control* trial*.mp. or random allocation/
2	Post- trial	(Post-trial or post trial or attrition or drop out? or dropout? or follow-up or followup? or extension* or trial closure? or long-term or longterm or extended observation* or extended stud*).mp.
3	Outcomes	(cost effective* or cost benefit* or costs or survivors or hospital admission? or hospitali?ation? or primary outcome* or secondary outcome* or primary endpoint? or secondary endpoint? or composite endpoint? or primary end point? or secondary end point? or composite end point? or outcome measure* or outcome assessment or outcomes research).mp. or treatment outcome/
4	Types of methodological follow-up	electronic data processings/or automatic data processing/or “surveys and questionnaires”/or telephone/or interview/or data collection methods/or data collection/or data linkage/or data system?.mp. or data reporting/or epidemiologic method?.mp. or incidence.mp. or mortality.mp. or health episode statistics.mp. or electronic health record?.mp. or electronic patient record?.mp. or computeri?ed record-linkage system?.mp. or national register*.mp. or national database*.mp. or episode of care/or routine data.mp. or routinely collected data.mp.
5		1 and 2 and 3 or 4
6	5	exp animals/not humans.sh.
7	6	limit to (abstracts and English language and yr=“2006 -Current”)

Key to operators used in MEDLINE/Ovid: where.pt is publication type, (?) represents any single character, (*) is a group of characters,.mp.is multi-purpose search,/is Medical Subject Headings (MeSH), exp is explode subject heading,.sh. is subject heading, (“ “) is phrase search.

#### EMBASE search strategy

- Search conducted via OvidSP interface 1974–2016, March 04

**Table:**

Step	Search terms
1	exp medical record/or interview/or telephone interview/or survey?.mp. or questionnaire?.mp. or data system?.mp. or epidemiologic method?.mp. or incidence.tw. or mortality.tw. or cardiovascular mortality/or cancer mortality/or *mortality/or health episode statistics.mp. or electronic health record?.mp. or electronic patient record?.mp. or computeri?ed record-linkage system?.mp. or national registr*.mp. or national database*.mp. or routine data.mp. or routinely collected data.mp.
2	(cost effective* or cost benefit* or costs).mp. or clinical effectiveness/or effectiveness.mp. or survivors.mp. or hospital admission?.mp. or hospital episode?.mp. or hospitali?ation?.mp. or primary outcome*.mp. or secondary outcome*.mp. or outcome measure*.mp. or outcome assessment.mp. or outcomes research.mp. or treatment outcome/or treatment duration/
3	randomized controlled trial/or ((random* or blind* or placebo*).tw. and major clinical study/)
4	(Post-trial or posttrial or attrition or drop out? or dropout? or follow-up or followup? or extension* or trial closure? or long-term or longterm or extended observation*).mp.
5	1 and 2 and 3 and 4
6	(exp animals/or nonhuman/) not human/
7	5 not 6
8	limit 7 to (english language and yr=“2000 -Current”)
9	(letter or editorial or conference*).pt.
10	8 not 9

#### Cochrane Library search strategy

- Search conducted via Cochrane Library via Wiley interface
- Cochrane Central Register for Controlled Trials *(Issue 2 of 12, February 2016)*
- Cochrane Methods Register *(Issue 3 of 4, July 2012)*

**Table:**

Step	Search terms
1	MeSH descriptor: [Surveys and Questionnaires] this term only
2	MeSH descriptor: [Data Collection] this term only
3	MeSH descriptor: [Interview] explode all trees
4	MeSH descriptor: [Automatic Data Processing] this term only
5	data collection or “data processing*” or “data system*” or “data linkage” or “data reporting” or incidence or mortality or “health episode statistics” or “electronic health record*” or “electronic patient record*” or “record linkage system” or “record-linkage system*” or “national registr*” or “national database” or “episode of care” or “routine data” or “routinely collected data”:ti,ab,kw (Word variations have been searched)
6	1 or 2 or 3 or 4 or 5
7	cost effective* or “cost benefit*” or costs or “clinical effectiveness” or survivors or “hospital admission*” or hospitalisation* or hospitalization* or “primary outcome*” or “secondary outcome*” or “outcome measure*” or “outcome assessment” or “outcomes research” or “treatment outcome”:ti,ab,kw (Word variations have been searched)
8	post trial or posttrial or attrition or “drop out*” or dropout* or “follow up” or followup* or extension or “trial closure*” or “long term” or longterm or “extended observation*”:ti,ab,kw (Word variations have been searched)
9	6 and 7 and 8

#### Clinical Trials search strategy

- https://clinicaltrials.gov/

**Table:**

	Search terms	Limits
1	post-trial OR “post trial” OR posttrial OR attrition OR “drop out” OR dropout OR extension OR extended	Studies With Results Interventional Studies
2	longterm OR long-term OR “long term” OR follow-up OR “follow up” OR followup	Studies With Results Interventional Studies
	1 or 2	

Comments:

All results will be downloaded with all fields displayed and in a tab delimited format. This file will then be opened in ExCel. Duplicates will be removed. The spreadsheet sort order will be changed to enrollment A-Z and studies with fewer than 1000 enrolees will be removed.

## Additional file

Additional file 1: PRISMA-P (Preferred Reporting Items for Systematic Review and Meta-Analysis Protocols) 2015 checklist: recommended items to address in a systematic review protocol: recommended items to address in a systematic review protocol. (DOC 85 kb)

## Abbreviations

CENTRAL: Cochrane Central Register of Controlled Trials
EMBASE: Excerpta Medica database
GRADE: Grading of Recommendations Assessment, Development and Evaluation
HES: Health Episode Statistics
HSCIC: Health and Social Care Information Centre
ICD-codes: International Classification of Diseases codes
PRISMA-P: Preferred Reporting Items for Systematic Review Protocols
PTFU: Post-trial follow-up
RCT: Randomized controlled trial

## References

[1] Bulbulia R, Bowman L, Wallendszus K, Parish S, Armitage J, Peto R, Collins R, Collins R, Meade T, Sleight P, Armitage J, Parish S, Peto R, Youngman L, Buxton M, De Bono D, George C, Fuller J, Keech A, Mansfield A, Pentecost B, Simpson D, Warlow C, McNamara J, O’Toole L, Doll R, Wilhelmsen L, Fox KM, Hill C, Sandercock P (2011). Effects on 11-year mortality and morbidity of lowering LDL cholesterol with simvastatin for about 5 years in 20 536 high-risk individuals: a randomised controlled trial. Lancet.

[2] Ford I, Murray H, McCowan C, Packard CJ (2016). Long-term safety and efficacy of lowering low-density lipoprotein cholesterol with statin therapy 20-year follow-up of west of Scotland coronary prevention study. Circulation.

[3] Moher D, Schulz KF, Altman DG (2001). The CONSORT statement: revised recommendations for improving the quality of reports of parallel-group randomised trials. Lancet.

[4] Brueton VC, Tierney JF, Stenning S, Meredith S, Harding S, Nazareth I, Rait G (2014). Strategies to improve retention in randomised trials: a Cochrane systematic review and meta-analysis. BMJ Open.

[5] Barton J, Young A, Lay M (2015). Introduction of electronic data capture method using participant-completed online web-based follow up questionnaire in mail-based study achieves expected benefits and positive participant feedback. Trials.

[6] Scuffham P, Chaplin SLR (2003). Incidence and costs of unintentional falls in older people in the United Kingdom. J Epidemiol Community Heal.

[7] Rathbone J, Carter M, Hoffmann T, Glasziou P (2015). Better duplicate detection for systematic reviewers: evaluation of Systematic Review Assistant-Deduplication Module. Syst Rev.

[8] Moher D, Shamseer L, Clarke M, Ghersi D, Liberati A, Petticrew M, Shekelle P, Stewart LA (2015). Preferred Reporting Items for Systematic Review and Meta-Analysis Protocols (PRISMA-P) 2015 statement. Syst Rev.

