# Supplementary material for: Post-trial follow-up methodology in large randomized controlled trials: a systematic review protocol
Source: Syst Rev. 2016 Dec 15;5:214. doi: 10.1186/s13643-016-0393-3 (PMC5159967; doi:10.1186/s13643-016-0393-3)
Supplement: Additional file 1: — PRISMA-P (Preferred Reporting Items for Systematic Review and Meta-Analysis Protocols) 2015 checklist: recommended items to address in a systematic review protocol: recommended items to address in a systematic review protocol. (DOC 85 kb) [file 13643_2016_393_MOESM1_ESM.doc]

**PRISMA-P (Preferred Reporting Items for Systematic review and Meta-Analysis Protocols) 2015 checklist: recommended items to address in a systematic review protocol***

| Section and topic | Item No | Checklist item |
| --- | --- | --- |
| ADMINISTRATIVE INFORMATION | | |
| Title: |  |  |
| Identification | 1a | Post-trial follow-up methodology in large randomised controlled trials: a systematic review protocol |
| Update | 1b | Not applicable |
| Registration | 2 | Not registered as not a health care intervention (not eligible for PROSPERO) |
| Authors: |  |  |
| Contact | 3a | 1. Rebecca Llewellyn-Bennett *(corresponding author),* University of Oxford [rebecca.llewellyn-bennett@ndph.ox.ac.uk](mailto:rebecca.llewellyn-bennett@ndph.ox.ac.uk)   Clinical Trial Service Unit (CTSU), Nuffield Department of Population Health, Richard Doll Building, Roosevelt Drive, Oxford OX3 7LF   1. Louise Bowman, University of Oxford [louise.bowman@ndph.ox.ac.uk](mailto:louise.bowman@ndph.ox.ac.uk) 2. Richard Bulbulia, University of Oxford [richard.bulbulia@ndph.ox.ac.uk](mailto:richard.bulbulia@ndph.ox.ac.uk) |
| Contributions | 3b | Rebecca Llewellyn-Bennett: Writer of protocol and lead for systematic review design and initiation.  Louise Bowman: Reviewer and supervisor of systematic review and protocol (guarantor of the review)  Richard Bulbulia: Reviewer and supervisor of systematic review and protocol |
| Amendments | 4 | This protocol does not represent an amendment of a previously completed or published protocol |
| Support: |  |  |
| Sources | 5a | CTSU, Nuffield Department of Population Health, University of Oxford, United Kingdom |
| Sponsor | 5b | CTSU, Nuffield Department of Population Health, University of Oxford, United Kingdom |
| Role of sponsor or funder | 5c | Collaboration and support from CTSU with advice and expertise |
| INTRODUCTION | | |
| Rationale | 6 | Randomised controlled trials (RCTs) are the gold standard for investigating an intervention. However RCTs are costly and usually employ a brief treatment period with limited in-trial follow-up. Post- trial follow-up can detect persistent treatment effects, safety issues or enhanced benefits that were not apparent during in-trial. However, there is no consensus on optimal methodology for post-trial follow-up. |
| Objectives | 7 | The design of this protocol is to qualitatively compare post-trial follow-up methodologies used in large randomised controlled trials. We intend to review retention rates of participants and cost-effectiveness of post-trial follow-up where information is available. |
| METHODS | | |
| Eligibility criteria | 8 | All published large randomized controlled trials of >1000 participants which had post-trial follow-up published between 2006- 2016. The title and abstract must be in English.(Table 1) |
| Information sources | 9 | From February 2006 - February 2016. Electronic databases will include Cochrane methodology group register, Cochrane Central Register of Controlled Trials (CENTRAL), Medline, Embase and trials registries (clinical-trials.gov) |
| Search strategy | 10 | Table 2 for MESH and defined keywords |
| Study records: |  |  |
| Data management | 11a | All search strategies will be recorded in a Excel spreadsheet recording the date and number of articles. Eligible studies from the search will be transferred into Endnote reference management software. Using Endnote, duplicates will be removed using the “deduplication tool”. |
| Selection process | 11b | See Figure 1 |
| Data collection process | 11c | See Figure 2 |
| Data items | 12 | Retention rates, missing data, costings, length of post-trial follow-up, numbers of participants followed-up compared to in-trial numbers |
| Outcomes and prioritization | 13 | 1. Retention rates of participants in post-trial follow-up 2. Cost-effectiveness by determinant of cost of post-trial follow-up 3. The quality of data gained e.g. amount of missing data if recorded |
| Risk of bias in individual studies | 14 | Cochrane Risk of Bias tool will used at the study level. |
| Data synthesis | 15a | Kappa statistics will be used for quantitatively assess for concordance between 2 authors when screening and extracting data. No other data synthesis planned. |
|  | 15b | Summarisation and comparison of heterogeneous data will be compared in tables |
|  | 15c | Cochrane Risk of Bias will be used. |
|  | 15d | Grade Tool for evidence |
| Meta-bias(es) | 16 | No planned assessment of meta-bias |
| Confidence in cumulative evidence | 17 | GRADE will be used |

*** It is strongly recommended that this checklist be read in conjunction with the PRISMA-P Explanation and Elaboration (cite when available) for important clarification on the items. Amendments to a review protocol should be tracked and dated. The copyright for PRISMA-P (including checklist) is held by the PRISMA-P Group and is distributed under a Creative Commons Attribution Licence 4.0.**

*From: Shamseer L, Moher D, Clarke M, Ghersi D, Liberati A, Petticrew M, Shekelle P, Stewart L, PRISMA-P Group. Preferred reporting items for systematic review and meta-analysis protocols (PRISMA-P) 2015: elaboration and explanation. BMJ. 2015 Jan 2;349(jan02 1):g7647.*
